# *Staphylococcus aureus* Isolates Encode Variant Staphylococcal Enterotoxin B Proteins That Are Diverse in Superantigenicity and Lethality

**DOI:** 10.1371/journal.pone.0041157

**Published:** 2012-07-16

**Authors:** Petra L. Kohler, Seth D. Greenwood, Suba Nookala, Malak Kotb, David M. Kranz, Patrick M. Schlievert

**Affiliations:** 1 Department of Microbiology, University of Minnesota Medical School, Minneapolis, Minnesota, United States of America; 2 Department of Molecular Genetics, Biochemistry, and Microbiology, University of Cincinnati Medical School, Cincinnati, Ohio, United States of America; 3 Department of Biochemistry, School of Molecular and Cellular Biology, University of Illinois, Urbana, Illinois, United States of America; 4 Department of Microbiology, University of Iowa Carver College of Medicine, Iowa City, Iowa, United States of America; University of Edinburgh, United Kingdom

## Abstract

*Staphylococcus aureus* produces superantigens (SAgs) that bind and cross-link T cells and APCs, leading to activation and proliferation of immune cells. SAgs bind to variable regions of the β-chains of T cell receptors (Vβ-TCRs), and each SAg binds a unique subset of Vβ-TCRs. This binding leads to massive cytokine production and can result in toxic shock syndrome (TSS). The most abundantly produced staphylococcal SAgs and the most common causes of staphylococcal TSS are TSS toxin-1 (TSST-1), and staphylococcal enterotoxins B and C (SEB and SEC, respectively). There are several characterized variants of humans SECs, designated SEC1-4, but only one variant of SEB has been described. Sequencing the *seb* genes from over 20 *S. aureus* isolates show there are at least five different alleles of *seb*, encoding forms of SEB with predicted amino acid substitutions outside of the predicted immune-cell binding regions of the SAgs. Examination of purified, variant SEBs indicates that these amino acid substitutions cause differences in proliferation of rabbit splenocytes *in vitro*. Additionally, the SEBs varied in lethality in a rabbit model of TSS. The SEBs were diverse in their abilities to cause proliferation of human peripheral blood mononuclear cells, and differed in their activation of subsets of T cells. A soluble, high-affinity Vβ-TCR, designed to neutralize the previously characterized variant of SEB (SEB1), was able to neutralize the variant SEBs, indicating that this high-affinity peptide may be useful in treating a variety of SEB-mediated illnesses.

## Introduction

*Staphylococcus aureus* produces an array of secreted virulence factors that enable the organism to colonize and cause illness in humans. These secreted virulence factors include the staphylococcal superantigens (SAgs). SAgs are defined by their abilities to bind and cross-link subsets of T cell receptors (TCRs) and major histocompatibility class II (MHC II) molecules on APCs in a location different than the traditional antigen-binding site, and explaining how SAgs are able to activate large numbers of T cells [reviewed in [1]]. SAgs recognize the variable portion of the TCR beta chain (Vβ-TCR), and each SAg recognizes a relatively unique subset of Vβ-TCRs [2], [3]. The binding and cross-linking of T cells and APCs by SAgs activates a large subset of T cells and APCs, leading to massive production of cytokines and the development of toxic shock syndrome (TSS) [2], [4]. SAgs are also associated with many other human illnesses in which TSS does not occur, possibly by locally affecting immune cells; SAgs have been shown to be involved in atopic dermatitis, purpura fulminans, extreme pyrexia, and necrotizing pneumonia [5], [6]. In addition, preliminary studies carried out in our laboratory showed that SAgs contribute to the formation of biofilms on heart valves during staphylococcal endocarditis, leading to the hallmark lesions termed vegetations [7].

Different staphylococcal isolates produce different SAgs with the most abundantly produced SAgs being TSS toxin-1 (TSST-1), staphylococcal enterotoxin B (SEB), and SEC. TSST-1 causes the majority of menstrual TSS cases and about half of non-menstrual TSS cases. SEB and SEC cause approximately half of non-menstrual TSS cases [8], [9], [10], [11], [12]. Most if not all staphylococcal strains designated as part of the CDC USA400 clonal group (by pulsed-field gel electrophoresis [PFGE]) produce large amounts of SEB or SEC. The USA400 PFGE clonal group are recently emergent and have been implicated in severe, fatal community-associated *S. aureus* infections [reviewed in [6]]. In 1999, four infants in the upper Midwest region of the United States developed fatal necrotizing pneumonia that was later shown to be caused by USA400 methicillin-resistant strains that produce SEB (2 strains) or SEC (2 strains) [13]. Strains in the USA400 clonal group have also been associated with skin infections, soft tissue infections, purpura fulminans, and post-viral TSS, and the production of SEB or SEC is highly associated with these illnesses [6].

The three-dimensional structure of SEB is nearly identical to that of SEC, and SEB is up to 69% identical to SEC in primary amino acid sequence [14], [15], [16]. There are several known variants of SEC, and four of these variants, designated SEC1, SEC2, SEC3, and SEC4 are associated with human *S. aureus* strains. The predicted amino acid sequences of the SECs are over 90% identical. The SECs are serologically cross-reactive but vary in their abilities to stimulate immune cells because of variation in the subsets of Vβ-TCRs that they bind [reviewed in [1]]. In contrast to the characterized varying forms of SEC, there is one previously characterized form of SEB, encoded by the sequenced *S. aureus* strain COL [17]. This SEB has been used for immunological and structural studies. We hypothesized, that because of primary sequence similarity among SEB and the SEC subtypes, there might be diversity among SEB sequences and differences in activities, similar to the diversity observed among SEC; this was tested in our studies.

## Materials and Methods

### Human blood

The collection and use of human blood (3 participants) was carried out in accordance with Institutional Review Board (IRB) approval. The University of Minnesota IRB specifically approved protocol 1004M80313 for the purpose of our studies in this manuscript. Written informed consent with signatures was obtained from all volunteers donating blood for these studies.

### Sequence analysis of variant alleles of *seb*

Genomic DNA was isolated from 20 clinical isolates of *S. aureus* and used as the template for amplification of the *seb* gene by PCR with the primers *seb*5′F3 (5′ –GTGTATCTAGATACTTTTTGGGAATCTTGG –3′) and *seb*3′R3 (5′– GGGGTTAATGCTAAATCAGAGAGATATAAC –3′). Predicted, translated amino acid sequences were determined using the program SeqBuilder (DNASTAR), and amino acid sequence alignments were made using the Clustal W method in the program Megalign (DNASTAR).

### Cloning and purification of variant SEBs

SEB from the strain MNHO was purified by isoelectric focusing as previously described [8]. The amino acid sequence of SEB from MNHO is predicted to be identical to SEB from strain COL, as indicated by sequencing the MNHO *seb* gene. SEB from strain MNBE was purified in an identical manner. Briefly, strains MNHO and MNBE were grown for two days in beef heart dialysate medium. Cells and secreted proteins were precipitated in ethanol, dried, and resuspended in water. The soluble molecules were separated by thin layer isoelectric focusing on gradients of pH 3 to 10. Proteins that focused in the pH range of 6 to 10 were refocused on gradients of pH 7 to 9. The gradients were fractionated, and double immunodiffusion with anti-SEB antibodies (Toxin Technologies, Sarasota, FL) was used to determine which fractions contained SEB. The concentrations of the toxins were determined by using the Biorad protein assay, and after lyophilization, the proteins were resuspended in water. Five micrograms of each of the toxins were separated by SDS-PAGE (12% acrylamide), and the gels were stained with Coomassie Brilliant Blue R250 to demonstrate the proteins had been purified to homogeneity.

SEB from strain MNBD could not be purified directly from MNBD because this strain unusually also produces SEC. SEC focuses to approximately the same pH as SEB, making it impossible to separate the toxins using our method of purification. To avoid this problem, the *seb* gene and its upstream promoter region from strain MNBD were amplified by PCR using the primers *sebBam*HIF (5′– GTATCGGGATCCGATGTTTTCGTATATAAGTTTAGGTGATGTATAG –3′) and *sebEco*RIR (5′– GTCAGTGAATTCCAACAAGGGGTTAATGCTAAATCAG –3′). Restriction sites in the primers are underlined. The resulting product was digested with *Eco*RI and *Bam*HI and ligated to the shuttle vector pCE104 that had been digested with the same restriction enzymes. The resulting ligation mixture was used to transform *Escherichia coli*, and transformants were selected by growing on LB agar containing 100 µg/ml ampicillin. Plasmids in the resulting transformants were screened by restriction digestion and sequencing to verify the sequence of the cloned *seb* gene. The resulting plasmid, pPLK23, was transformed to the *S. aureus* laboratory strain RN4220 by electroporation. Transformants were selected by growth on Todd-Hewitt agar with 5 µg/ml erythromycin. Plasmids from erythromycin resistant clones were screened by restriction digestion. The resulting strain of RN4220 carrying pPLK23 was grown for 2 days in beef heart dialysate medium with 5 µg/ml erythromycin. The secreted SEB protein was purified with ethanol precipitation and IEF as described above.

### Trypsin sensitivity assay

The trypsin sensitivity assay was carried out as described by Chi et al. with minor modifications [14]. Briefly, 20 μg of purified SEB was incubated with 100 ng/ml trypsin type XI (Sigma-Aldrich, St. Louis, MO) with 25 mM Tris-HCl and 20 mM CaCl_2_ in a 100 µl reaction volume at 37°C. Aliquots (10 µl) were removed from the trypsin digestion reactions at designated time points and immediately boiled for 5 min with 10 µl of 2X SDS-PAGE sample buffer. Control reactions identical to the trypsin digest reactions but lacking trypsin were incubated at 37°C for the duration of the assay after which aliquots of the control reactions were boiled in an identical manner to the trypsin digest reactions. The boiled samples were separated by SDS-PAGE on 16% acrylamide gels, and the gels were stained with Coomassie Brilliant Blue R250, destained, and photographed.

### Splenocyte proliferation assay

The splenocyte proliferation assay was carried out as previously described [18]. Briefly, rabbit spleens were harvested from euthanized animals and immediately placed in RPMI tissue culture medium supplemented with penicillin, streptomycin, heat inactivated fetal calf serum, and glutamine. Splenocytes were harvested from spleens, washed, counted, and diluted into complete RPMI at a concentration of 1.5×10^6^ cells/ml. Aliquots (200 µl) of the cell suspension was transferred into wells of a 96-well tissue culture dish. SEB SAgs were diluted in complete RPMI and added to the cells in 20 µl volumes. Splenocytes were incubated with SEBs for three days after which 1 µCi of ^3^H-thymidine was added to each well. The cells were allowed to incubate for an additional 24 h. The cells were harvested using a PHD vacuum cell harvester (Brandel, Gaithersburg, MD), and the amount of ^3^H-thymidine incorporated into the DNA of the cells was measured by liquid scintillation counting. To test the activity of anti-SEB high affinity Vβ-TCRs against variant SEBs, rabbit splenocytes were incubated with 1 µg of toxin, and varying concentrations of the anti-SEB Vβ-TCR designated G5-8. The proliferation of splenocytes was measured as described above.

### Human lymphocyte proliferation assay

Human peripheral blood mononuclear cells (PBMCs) were separated from venous blood by ficoll-hypaque density gradient centrifugation according to manufacturer's recommendations. The isolated PBMCs were added to wells in 96 well tissue culture plates in 200 µl volumes (1×10^6^ cells/ml) in complete RPMI medium. SEBs and high-affinity Vβ-TCR G5-8 were added to wells, and plates incubated and radiolabeled as previously described for rabbit splenocyte assays.

### Rabbit model of TSS

SAgs amplify the lethal effects of gram negative lipopolysaccharide (LPS) by up to millions of fold when administered intravenously to rabbits. It has been shown previously that this synergized lethality parallels the course of lethal TSS, except the time-course is accelerated such that lethal TSS occurs in 24–48 h after toxin administration [19]. Rabbits were administered varying doses of SEB intravenously from 0.0007 to 10 µg/kg, and fevers were monitored for 4 h. Subsequently, the animals were given intravenous LPS (10 µg/kg), and euthanized when they displayed symptoms of universally lethal TSS. Deaths were recorded over a 48 h period. LD_50_ of the variant SEBs was determined by the method of Reed and Muench [20]. Animal experiments were carried out with approval of the Institutional Animal Care and Use Committee at the University of Minnesota according to protocol 0908A71722.

### Analysis of the Vβ-TCR repertoire

Quantitative analyses of the preferential expansion of T lymphocytes with specific Vβ-TCRs in response to SEB variants was conducted by flow cytometry using the IO Test Beta Mark Vβ-TCR repertoire kit (Beckman Coulter, Fullerton, CA, USA). PBMC were incubated at 4×10^6^cells/2 ml RPMI-1640 with 10% fetal calf serum (heat inactivated and polymyxin B treated) complete medium and stimulated with the indicated SEB variants (100 ng/ml) or PHA (1 µg/ml). After 72 h, the cells were cultured for an additional 24 h in the presence of 10 U/ml recombinant human IL-2 to allow for the regeneration of modulated T cell receptors. The cells were then harvested, washed extensively with PBS containing 1% bovine serum albumin, and stained with different Vβ-TCR specific antibodies, according to the manufacturer's instructions. Allophycocyanin (APC) conjugated anti human CD3 antibody was used as an additional marker to enable proper gating on T lymphocytes only. The CD3-APC blastogenic cells were gated on, and simultaneous analysis of 3 Vβ-TCRs per tube was performed using a FACSCanto flow cytometer (Becton Dickinson, San Jose, CA, USA). Data were analyzed using FlowJo software (Tree Star, Inc. Ashland, OR, USA). A minimum of 30,000 cell events was acquired for the analysis. The experiments were carried out using PBMCs from two healthy blood donors. Since the percentage of Vβ-TCR subsets varies between blood donors and after PBMC stimulation by PHA, we expressed the results as ratios of the Vβ-TCR expansion induced by SEB from MNHO or its SEB from its variants relative to that by PHA with respect to the same individual. A particular Vβ-TCR showing a >1 fold increase in ratio of SEB/PHA was considered expanded.

## Results

### Clinical isolates of *S. aureus* encode variant SEBs

**Figure 1:**
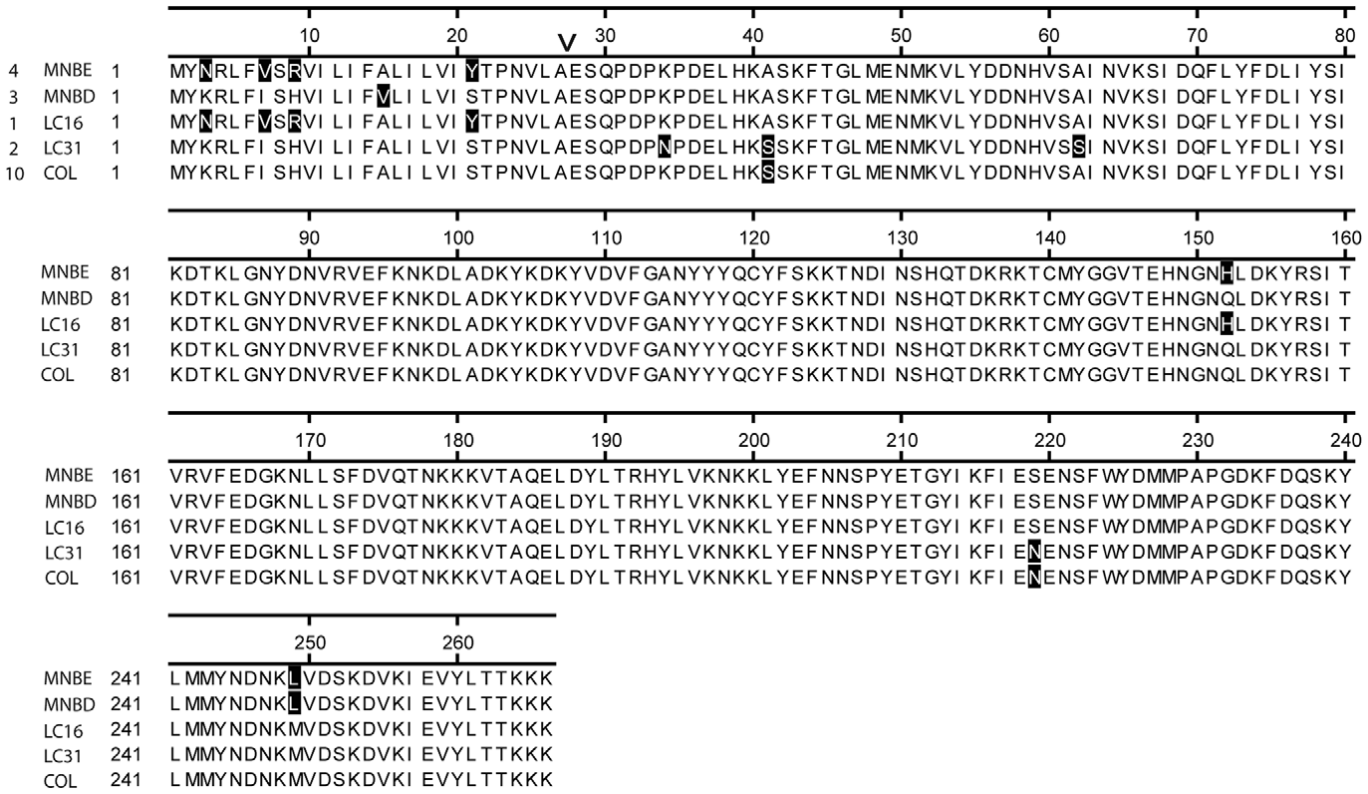
Alignment of the signal peptide and mature protein amino acid sequences of variant forms of SEB. The predicted, translated signal peptide and mature protein amino acid sequences of five variants of SEB were aligned by Clustal W. Varying amino acids are in red. Strain sequences listed are from MNBE, MNBD, LC16, LC31, and COL; the SEB sequences for strains MNHO and COL are identical. The numbers in front of strain names list the number of strains having that sequence. Numbers above the sequences indicate amino acid numbering including the signal peptide. Carat indicates the signal peptide cleavage site.

The *seb* gene of 20 clinical isolates of *S. aureus* was sequenced. Sequence alignment of the predicted amino acid sequences of the SEB proteins showed variation of the toxins among strains. Four strains contained the SEB sequence shown for MNBE, 3 for MNBD, 1 for LC16, 2 for LC31, and 10 for COL (including MNHO). Amino acid substitutions in SEB were identified in both the signal peptides and the secreted, active portions of the toxins (Fig. 1). The predicted signal peptides of SEBs encoded by the clinical isolates contained amino acid substitutions in five residues compared to the signal peptide of SEB encoded by strain MNHO. In total, there were six amino acid substitutions in the secreted (mature), active portions of the variant SEBs compared to the SEB encoded by MNHO (Fig. 1).

**Figure 2:**
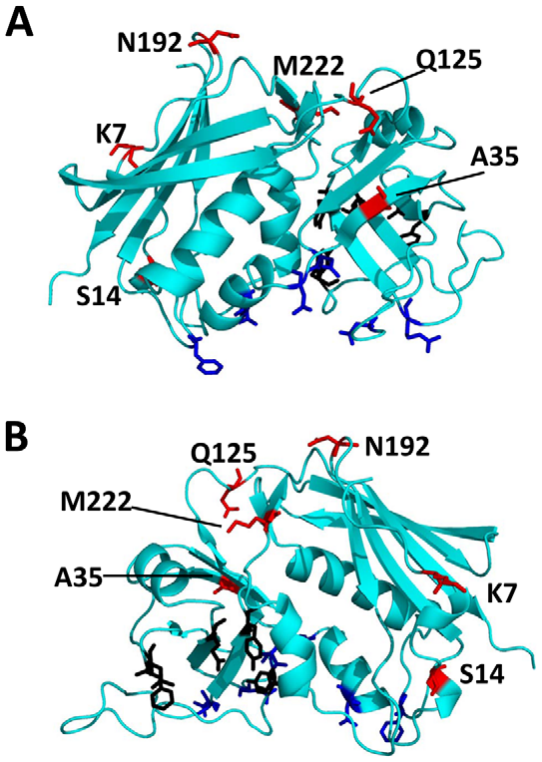
Amino acid substitutions in variants of SEB are not predicted to occur in regions that contact immune cell receptors. A and B are representations of the crystal structure of SEB shown from two different angles. Residues that contact the Vβ-TCR are highlighted in blue and residues that contact MHC II DR1 are highlighted in black. The amino acid substitutions found in variants of SEB are highlighted in red.

Based on the structure of SEB (MNHO) and its complexes with either TCR Vβ8 or MHC class II DR1, these amino acid substitutions (red in Fig. 2) are not predicted to occur in areas of the toxins that interact with the cellular receptors. These include key contact residues in the Vβ (N23, N31, N60, N88, R110, F177; blue in Fig. 2) or key contact residues in the class II MHC DR1 (F44, L45, E67, Y89, Y115; black in Fig. 2) [16], [21], [22], [23], [24], [25], [26], [27], [28].

We focused our studies on the variant SEBs encoded by strains MNBD and MNBE, compared to SEB encoded by MNHO/COL, because collectively these three strains encoded the majority of amino acid substitutions observed among variant SEBs in these clinical isolates (Fig. 1). The amino acid sequence of SEB from strain MNBE contains four amino acid substitutions in the secreted portion of the toxin when compared to the characterized variant of SEB produced by MNHO/COL (Fig. 1). The SEB encoded by strain MNBD is predicted to be identical to that of SEB from strain MNBE except for the amino acid at position 125 of the mature, secreted protein, which is a glutamine in the SEB encoded by MNBD. In contrast, this residue is a histidine in SEB encoded by strains MNBE and LC16. The crystal structures of SEC2 and SEC3 indicate that this conserved histidine, along with several other residues, coordinates a zinc ion in these toxins and are linked to the promotion of dimer formation [14], [15]. In addition, mutation of histidine 122 in SECs affects fever production in a rabbit model of TSS, and to cause emesis in a feline model [14], [29].

### Variants of SEB differ in their ability to cause proliferation of rabbit splenocytes

We investigated the activity of the SEBs encoded by strains MNHO (identical to COL), MNBE and MNBD. The purified SEBs were digested with trypsin to examine their stability to trypsin as a measure of whether the three toxin preparations had a similar fraction properly folded, or that they have a significant difference in domain stability. All three preparations of SEB exhibited similar patterns of digestion by trypsin over time (Fig. S1). This result indicates that amino acid substitutions in the SEBs do not grossly affect the overall stability or folding of the toxins. Analysis by inductively coupled plasma mass spectrometry (ICP-MS) indicated that the purified SEBs contained no zinc or other metal ions (data not shown).

**Figure 3:**
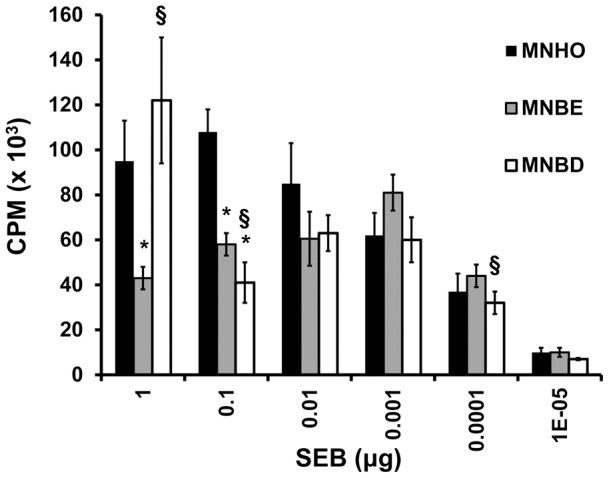
Variant SEBs differ in their ability to cause proliferation of rabbit splenocytes. Rabbit splenocytes were exposed to varying concentrations of SEB from strains MNBE, MNBD, or MNHO, and subsequently incubated with ^3^H-thymidine. Cellular proliferation is reported as the measure of the counts per minute of ^3^H-thymidine that had incorporated into the DNA of the splenocytes. The mitogenicity of each toxin was measured in quadruplicate. The three toxins caused different patterns of splenocyte proliferation. Error bars represent the standard deviations, and statistical significance was determined by using Student's unpaired *t*-test. *, *P*<0.05 with respect to MNHO. §, *P*<0.05 with respect to MNBE.

The purified SEBs were assayed for superantigencity. Doses of 1 µg or 0.1 µg of SEB from strain MNBE caused significantly less proliferation of splenocytes than identical doses of the variant of SEB encoded by strain MNHO (*P*<0.002) (Fig. 3). In contrast, 1 µg of SEB encoded by strain MNBD caused proliferation of splenocytes that was similar to that of 1 µg SEB encoded by MNHO, but 1 µg of SEB from MNBD caused significantly more proliferation of splenocytes than 1 µg SEB from MNBE (*P*<0.002) (Fig. 3). A dose of 0.1 µg of SEB from strain MNBD caused significantly less proliferation of splenocytes than 0.1 µg of SEB from MNHO or MNBE (*P*<0.0001 and *P*<0.02 respectively). Doses of 0.001 µg and 0.0001 µg of SEB from MNBD caused significantly less proliferation of splenocytes than identical doses of SEB from MNBE (*P*<0.05 for 0.001 µg and *P*<0.008 for 0.0001 µg) (Fig. 3). Thus, amino acid changes in variant forms of SEB affect the ability of these toxins to cause proliferation of immune cells.

### Amino acid substitutions in variant SEBs affect lethality in a rabbit model of TSS

The pyrogenicity and lethality of variant SEBs were tested. The maximum dose of every SEB (10 µg/kg) administered to rabbits was comparably pyrogenic (data not shown). The dose of SEB from MNHO that is lethal for 50% of rabbits (LD_50_) was calculated to be 0.007 µg/kg (Table 1). This is nearly 10-fold lower than the calculated LD_50_ values for SEBs from MNBD and MNBE which were 0.074 µg/kg and 0.049 µg/kg, respectively (Table 1).

**Table 1:** LD_50_ determination of variant SEBs.

SEB Toxin Administered	SEB Dose (µg/kg)	Rabbits Dead/Total Rabbits Tested	LD_50_ (µg/kg)
MNHO	0.0007	0/2	0.007
	0.007	1/2	
	0.07	4/4	
	0.7	4/4	
	7.0	4/4	
MNBD	0.007	0/2	0.074
	0.075	2/4	
	0.75	4/4	
	7.5	4/4	
MNBE	0.001	0/2	0.049
	0.01	1/2	
	0.098	2/4	
	0.98	4/4	
	9.8	4/4	

### Variant SEBs are diverse in superantigenicity, but can be neutralized by a single soluble high-affinity Vβ-TCR

**Figure 4:**
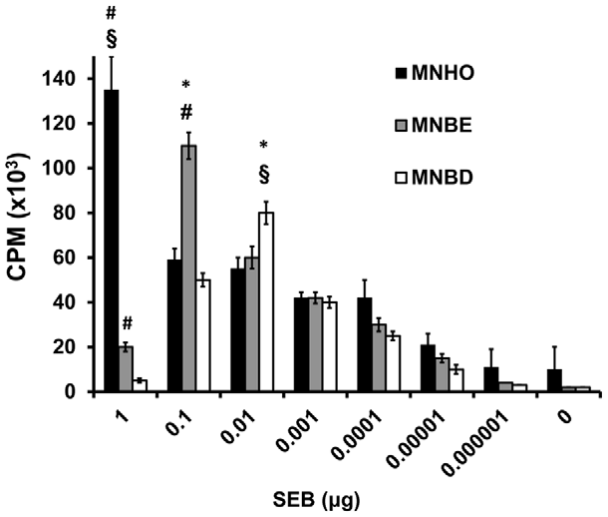
Variant SEBs differ in their abilities to cause proliferation of human lymphocytes. Human PBMCs were exposed to varying concentration of SEB from strains MNHO, MNBD, or MNBE, and subsequently incubated with ^3^H-thymidine. Cellular proliferation is reported as the measure of the counts per minute of ^3^H-thymidine that had incorporated into the DNA of the PBMCs. The mitogenicity of each toxin was measured in quadruplicate. The three toxins caused different patterns of monocyte proliferation. Error bars represent the standard deviations, and statistical significance was determined by using Student's unpaired *t*-test. *, *P*<0.05 with respect to MNHO; §*P*<0.05 with respect to MNBE; #, *P*<0.05 with respect to MNBD.

We next tested the ability of variant SEBs to cause proliferation of human lymphocytes. SEB (1 µg) from strain MNHO caused significantly more proliferation of lymphocytes than 1 µg of SEB from strains MNBD or MNBE (*P*<0.05) (Fig. 4). In contrast, 0.1 µg of SEB from strain MNBD caused significantly more proliferation of lymphocytes than 0.1 µg of SEB from strains MNBD or MNHO (Fig. 4). Amounts of toxin below 0.01 µg caused equal proliferation of lymphocytes among the variant SEB types (Fig. 4). These patterns of varying superantigenicity with different concentrations of toxin suggest that the variant SEBs bind different subsets of Vβ-TCRs or bind TCRs with different affinities.

**Figure 5:**
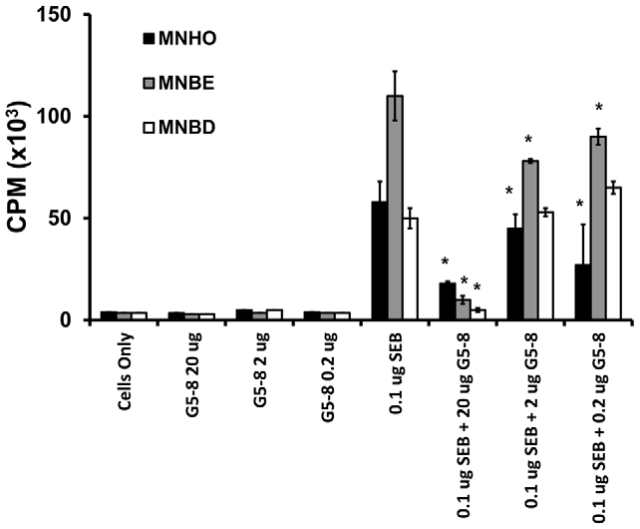
Variant SEB superantigenicity is neutralized by a soluble, high-affinity Vβ-TCR protein. Human PBMCs were exposed to SEB and varying concentrations of the G5-8 high affinity Vβ-TCR protein, and subsequently incubated with ^3^H-thymidine. Cellular proliferation is reported as the measure of the counts per minute of ^3^H-thymidine that had incorporated into the DNA of the PBMCs. Error bars represent the standard deviations, and statistical significance was determined by using Student's unpaired *t*-test. *, *P*<0.05 for statistically significant differences with respect to toxin alone.

Next, we tested the ability of a soluble high-affinity Vβ-TCR that binds SEB to neutralize proliferation of PBMCs. The soluble Vβ-TCR, designated G5-8, was engineered to bind with 3×10^6^ higher affinity than its parent Vβ8-TCR to the previously characterized variant of SEB from strains COL/MNHO and neutralizes SEB *in vitro* and in the rabbit model of TSS, eliminating the superantigenicity of the toxin [30]. Addition of 20 µg of G5-8 to any of the variant SEBs in the lymphocyte proliferation assay significantly reduced the proliferation of PBMCs for all forms of SEB tested (*P<*0.05 compared to toxin alone) (Fig. 5). As the amount of G5-8 was reduced, there was less attenuation of proliferation induced by each of the SEBs (Fig. 5). Addition of G5-8 alone resulted in no proliferation of PBMCs (Fig. 5). G5-8 is thus able to neutralize all forms of SEB tested.

### Human Vβ-TCR skewing of the variant forms of SEB

**Figure 6:**
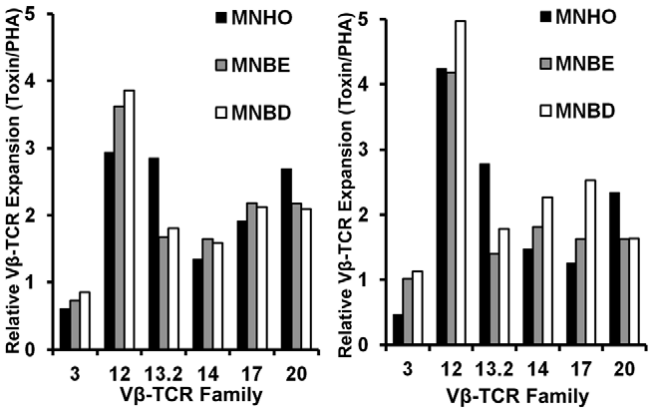
There is diversity in the profile of T-cell receptors that are bound by variant SEBs. We compared the Vβ-TCR expansion in PHA stimulated and SEB variants stimulated PBMC. In all, 24 Vβ-TCRs were analyzed. APC conjugated CD3^+^ blastogenic cells were gated on and simultaneous analysis of 3 Vβ-TCRs per tube was performed. The results are expressed as ratios of the Vβ-TCR expansion induced by SEB from MNHO or SEB from its variants relative to that by PHA with respect to the same individual. Data are shown from two different donor samples. Vβ-TCRs showing a >1 fold increase in ratio of SEB/PHA were considered expanded.

SAgs display a distinctive ability to cause expansion of T cells expressing specific Vβ-TCR populations, and each SAg can be characterized by its Vβ-TCR “signature”. The Vβ-TCR “signature” for the characterized variant of SEB produced by MNHO/COL can be detected by the preferential expansion of T cells expressing Vβ3, Vβ12, Vβ13.2, Vβ14, Vβ17 and Vβ20 [31]. Since the percentage of TCR Vβ subsets varies between blood donors and after PBMC stimulation by PHA, we expressed the results as ratios of the TCR Vβ expansion induced by SEB from MNHO or SEB from its variants relative to that by PHA with respect to the same individual. The expansion of Vβ3, Vβ12, Vβ14 and Vβ17 was similar in response to all three SEB variants (Fig. 6); however, the characterized variant of SEB (MNHO) consistently elicited more expansion of T cells expressing Vβ13.2 and Vβ20 than variant SEB produced by MNBE or MNBD (Fig. 6).

## Discussion

*S. aureus* produces a variety of secreted virulence factors, including cytolysins that alter immune function in the local infection environment and SAgs that systemically alter immune system function through their effects of T lymphocyte and APC cytokine production [reviewed in [32]]. The end results of SAg action are serious human illnesses, such as TSS.

Staphylococcal SAgs are a large family of proteins whose functions are well-characterized. These include SEs A–E and SEI, SE-like H and J–X, and TSST-1. Of these superantigens, three are the primary toxins associated with human illnesses: TSST-1 and SEs B and C [reviewed in [1]]. Through our studies of hundreds of strains of human *S. aureus* producing TSST-1, we have identified only one form, TSST-1 Human. However, a second form of TSST-1 has been described, TSST-Ovine that differs by 7 amino acids from TSST-1 Human. TSST-Ovine is biologically inactive as a superantigen when exposed to human T cells but is active in stimulation of T cells from sheep [33]. Four major subtypes of SEC have been identified, designated SEC1, SEC2, SEC3, and SEC4. These four subtypes retain ability to react with antisera to SEC, but each has subtype specific epitopes. Additionally, the four subtypes differ in their Vβ-TCR usage profiles, and these differences may lead to differences in biological toxicity [reviewed in [1]].

Because of the similarity of SEC to SEB, we hypothesized that SEB may exist as subtypes, comparable to SEC. This study demonstrated the existence of multiple SEB subtypes, some of which have been shown to have alterations in biological activities. Of the 20 strains sequenced, the majority of SEBs were SEB1, the SEB present in well-characterized strains such as COL and MNHO. Among the variant SEBs, there were five predicted amino acid substitutions in the signal peptide region of the proteins when the predicted amino acid sequences to the SEB encoded by strains COL and MNHO. In addition, there were six predicted amino acid substitutions in the mature, secreted portion of the proteins. In total, sequencing 20 strains revealed four predicted SEB sequences with amino acid substitutions compared to the SEB encoded by strains COL and MNHO. Based on our findings that 20% of clinical isolates tested have variant forms of SEB, it is likely that additional variant SEBs will be identified in the future. However, these proteins are likely to be selected to maintain sufficient sequence identity to preserve immunobiological activity.

We chose to focus on three SEB subtypes for further analysis. These were encoded by strains MNBD, MNBE, and MNHO. The mature, secreted form of the SEBs encoded by MNBE and MNBD are predicted to differ from the SEB produced by MNHO by four and three amino acids, respectively. MNBE and MNBD are predicted to differ from each other by only one amino acid at position 152. The predicted histidine at position 152 of the SEB encoded by MNBE was of particular interest because a histidine is conserved at this position in the SECs. This histidine in the SECs is known to coordinate a metal ion and we hypothesized that it could be important for the function of the variant SEBs.

ICP analysis, however, indicated that the variant SEB produced by strain MNBE does not contain any metals. The SEBs from strains MNBD, MNBE, and MNHO were purified and analyzed further. Although the amino acid substitutions in the variant SEBs are not predicted to be in parts of the toxin that interact with immune cells, we hypothesized that these substitutions may affect the lethality or superantigenicity of the toxins.

Our experiments revealed that the subtle changes in the amino acid sequences of the variant SEBs affected immune cell proliferation caused by the toxins. We examined the proliferation of rabbit splenocytes, as well as human PBMCs, induced by exposure to the variant SEBs. The pattern of immune cell proliferation was different for each SEB. At high concentrations (1 or 0.1 µg), the SEBs differed in their patterns of inducing proliferation of immune cells. Interestingly, at some concentrations, immune cell proliferation caused by SEBs from MNBE and MNBD differed. This is striking because these SEBs are predicted to differ only by one amino acid at position 152 of the mature, secreted toxin.

We also investigated the Vβ-TCR types of human cells that proliferated in response to exposure to the variant SEBs. Interestingly, the SEB produced by MNHO caused a consistently large expansion of cells expressing Vβ-TCR 13.2. This expansion was greater than that caused by exposure to SEBs produced by MNBD or MNBE for both donor blood samples tested. Vβ-TCR 13.2 is known to respond to SEB, but the amino acid substitutions predicted to occur in SEBs from MNBE and MNBD are not predicted to occur in sites that interact with this Vβ-TCR. The difference in proliferation of Vβ-TCR 13.2-expressing immune cells after exposure to variant SEBs is surprising given that that SEBs vary by a maximum of three amino acids that are not predicted to interact with the TCR.

In addition to *in vitro* investigations of lymphocyte proliferation caused by SEB, we investigated the lethality of the variant SEBs in a rabbit model of TSS. The calculated dose of SEB required to cause lethality in 50% of rabbits was nearly 10-fold lower for SEB from MNHO than the SEBs produced by MNBD or MNBE. Based on models of SEB interaction with immune cell receptors, it would not be predicted that the substituted amino acids in the variant SEBs would affect lethality in the rabbit model of TSS, so the results of these experiments were surprising. Together with the results of *in vitro* proliferation assays, these results suggest that subtle changes in the amino acid sequence of the toxin could affect folding and the ability of the toxins to interact with TCRs.

Of high importance is the finding that the soluble high-affinity Vβ-TCR that we have shown neutralizes SEB1, also neutralizes other forms of SEB and with the same approximate activity. This neutralization likely results from all SEBs sharing Vβ-TCR 8 binding with which the high-affinity Vβ-TCR G5-8, used in our studies, interacts.

In sum, our studies have shown that, like SEC, there are multiple subtypes of SEB with differences in biological activities. However, all subtypes tested can be neutralized by the same Vβ-TCR G5-8. These studies suggest that the Vβ-TCR G5-8 could be used to neutralize SEB in humans with illnesses caused by a variety of SEB variants.

## Supporting Information

Figure S1 **Variant SEBs show identical trypsin-digestion patterns.** Purified SEBs were digested with trypsin, and samples were analyzed by SDS-PAGE and Coomassie staining over time. A, MNHO; B, MNBD; C, MNBE.
(TIF)

## References

[1] McCormick JK, Yarwood JM, Schlievert PM (2001). Toxic shock syndrome and bacterial superantigens: an update.. Annu Rev Microbiol.

[2] Marrack P, Kappler J (1990). The staphylococcal enterotoxins and their relatives.. Science.

[3] Kappler J, Kotzin B, Herron L, Gelfand EW, Bigler RD (1989). V beta-specific stimulation of human T cells by staphylococcal toxins.. Science.

[4] Kotzin BL, Leung DY, Kappler J, Marrack P (1993). Superantigens and their potential role in human disease.. Adv Immunol.

[5] Schlievert PM, Case LC, Strandberg KL, Abrams BB, Leung DY (2008). Superantigen profile of *Staphylococcus aureus* isolates from patients with steroid-resistant atopic dermatitis.. Clin Infect Dis.

[6] Schlievert PM, Strandberg KL, Lin YC, Peterson ML, Leung DY (2010). Secreted virulence factor comparison between methicillin-resistant and methicillin-sensitive *Staphylococcus aureus*, and its relevance to atopic dermatitis.. J Allergy Clin Immunol.

[7] Pragman AA, Yarwood JM, Tripp TJ, Schlievert PM (2004). Characterization of virulence factor regulation by SrrAB, a two-component system in *Staphylococcus aureus*.. J Bacteriol.

[8] Schlievert PM, Shands KN, Dan BB, Schmid GP, Nishimura RD (1981). Identification and characterization of an exotoxin from *Staphylococcus aureus* associated with toxic-shock syndrome.. J Infect Dis.

[9] Schlievert PM, Tripp TJ, Peterson ML (2004). Reemergence of staphylococcal toxic shock syndrome in Minneapolis-St. Paul, Minnesota, during the 2000–2003 surveillance period.. J Clin Microbiol.

[10] Schlievert PM (1986). Staphylococcal enterotoxin B and toxic-shock syndrome toxin-1 are significantly associated with non-menstrual TSS.. Lancet.

[11] Crass BA, Bergdoll MS (1986). Involvement of staphylococcal enterotoxins in nonmenstrual toxic shock syndrome.. J Clin Microbiol.

[12] Bergdoll MS, Crass BA, Reiser RF, Robbins RN, Davis JP (1981). A new staphylococcal enterotoxin, enterotoxin F, associated with toxic-shock-syndrome *Staphylococcus aureus* isolates.. Lancet.

[13] CDC (1999). From the Centers for Disease Control and Prevention. Four pediatric deaths from community-acquired methicillin-resistant *Staphylococcus aureus*–Minnesota and North Dakota, 1997–1999.. JAMA.

[14] Chi YI, Sadler I, Jablonski LM, Callantine SD, Deobald CF (2002). Zinc-mediated dimerization and its effect on activity and conformation of staphylococcal enterotoxin type C. J Biol Chem.

[15] Papageorgiou AC, Acharya KR, Shapiro R, Passalacqua EF, Brehm RD (1995). Crystal structure of the superantigen enterotoxin C2 from *Staphylococcus aureus* reveals a zinc-binding site.. Structure.

[16] Papageorgiou AC, Tranter HS, Acharya KR (1998). Crystal structure of microbial superantigen staphylococcal enterotoxin B at 1.5 A resolution: implications for superantigen recognition by MHC class II molecules and T-cell receptors.. J Mol Biol.

[17] Yarwood JM, McCormick JK, Paustian ML, Orwin PM, Kapur V (2002). Characterization and expression analysis of *Staphylococcus aureus* pathogenicity island 3. Implications for the evolution of staphylococcal pathogenicity islands.. J Biol Chem.

[18] Poindexter NJ, Schlievert PM (1985). Toxic-shock-syndrome toxin 1-induced proliferation of lymphocytes: comparison of the mitogenic response of human, murine, and rabbit lymphocytes.. J Infect Dis.

[19] Schlievert PM (1982). Enhancement of host susceptibility to lethal endotoxin shock by staphylococcal pyrogenic exotoxin type C. Infect Immun.

[20] Reed LJ, Muench H (1938). A simple method of estimating fifty percent endpoints.. Am J Hyg.

[21] Baker MD, Papageorgiou AC, Titball RW, Miller J, White S (2002). Structural and functional role of threonine 112 in a superantigen *Staphylococcus aureus* enterotoxin B. J Biol Chem.

[22] Kappler JW, Herman A, Clements J, Marrack P (1992). Mutations defining functional regions of the superantigen staphylococcal enterotoxin B. J Exp Med.

[23] Komisar JL, Small-Harris S, Tseng J (1994). Localization of binding sites of staphylococcal enterotoxin B (SEB), a superantigen, for HLA-DR by inhibition with synthetic peptides of SEB.. Infect Immun.

[24] Garcia C, Briggs C, Zhang L, Guan L, Gabriel JL (1998). Molecular characterization of the putative T-cell receptor cavity of the superantigen staphylococcal enterotoxin B. Immunology.

[25] Briggs C, Garcia C, Zhang L, Guan L, Gabriel JL (1997). Mutations affecting the superantigen activity of staphylococcal enterotoxin B. Immunology.

[26] Neill RJ, Jett M, Crane R, Wootres J, Welch C (1996). Mitogenic activities of amino acid substitution mutants of staphylococcal enterotoxin B in human and mouse lymphocyte cultures.. Infect Immun.

[27] Jardetzky TS, Brown JH, Gorga JC, Stern LJ, Urban RG (1994). Three-dimensional structure of a human class II histocompatibility molecule complexed with superantigen.. Nature.

[28] Li H, Llera A, Tsuchiya D, Leder L, Ysern X (1998). Three-dimensional structure of the complex between a T cell receptor beta chain and the superantigen staphylococcal enterotoxin B. Immunity.

[29] Wang X, Zhang H, Xu M, Cai Y, Liu C (2009). Biological characterization of the zinc site coordinating histidine residues of staphylococcal enterotoxin C2.. Microbiology.

[30] Buonpane RA, Churchill HR, Moza B, Sundberg EJ, Peterson ML (2007). Neutralization of staphylococcal enterotoxin B by soluble, high-affinity receptor antagonists.. Nat Med.

[31] Thomas D, Dauwalder O, Brun V, Badiou C, Ferry T (2009). *Staphylococcus aureus* superantigens elicit redundant and extensive human Vbeta patterns.. Infect Immun.

[32] Brosnahan AJ, Schlievert PM (2011). Gram-positive bacterial superantigen outside-in signaling causes toxic shock syndrome.. FEBS J.

[33] Lee PK, Kreiswirth BN, Deringer JR, Projan SJ, Eisner W (1992). Nucleotide sequences and biologic properties of toxic shock syndrome toxin 1 from ovine- and bovine-associated *Staphylococcus aureus*.. J Infect Dis.

