# Supplementary figures and images for: Staphylococcus aureus Isolates Encode Variant Staphylococcal Enterotoxin B Proteins That Are Diverse in Superantigenicity and Lethality
Source: PLoS One. 2012 Jul 16;7(7):e41157. doi: 10.1371/journal.pone.0041157 (PMC3397982; doi:10.1371/journal.pone.0041157)

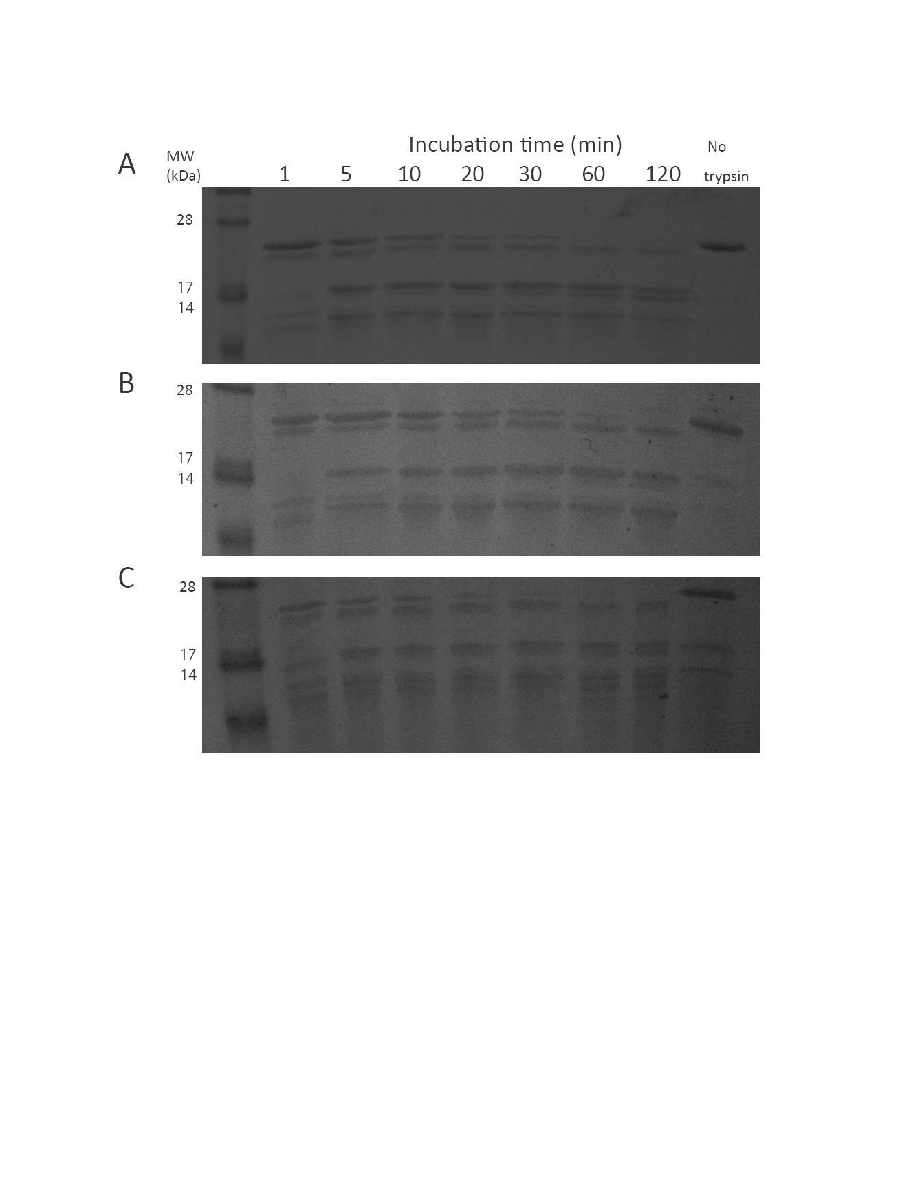

Supplement: Figure S1 — Variant SEBs show identical trypsin-digestion patterns. Purified SEBs were digested with trypsin, and samples were analyzed by SDS-PAGE and Coomassie staining over time. A, MNHO; B, MNBD; C, MNBE. (TIF) [file pone.0041157.s001.tif]
